# Integrated Microfluidic Nucleic Acid Isolation, Isothermal Amplification, and Amplicon Quantification

**DOI:** 10.3390/microarrays4040474

**Published:** 2015-10-20

**Authors:** Michael G. Mauk, Changchun Liu, Jinzhao Song, Haim H. Bau

**Affiliations:** School of Engineering and Applied Sciences, University of Pennsylvania, 220 S 33 rd Street, Philadelphia, PA 19104, USA; E-Mails: lchangc@seas.upenn.edu (C.L.); songjinz@seas.upenn.edu (J.S.); bau@seas.upenn.edu (H.H.B.)

**Keywords:** microfluidics, lab on a chip, isothermal nucleic acid amplification

## Abstract

Microfluidic components and systems for rapid (<60 min), low-cost, convenient, field-deployable sequence-specific nucleic acid-based amplification tests (NAATs) are described. A microfluidic point-of-care (POC) diagnostics test to quantify HIV viral load from blood samples serves as a representative and instructive example to discuss the technical issues and capabilities of “lab on a chip” NAAT devices. A portable, miniaturized POC NAAT with performance comparable to conventional PCR (polymerase-chain reaction)-based tests in clinical laboratories can be realized with a disposable, palm-sized, plastic microfluidic chip in which: (1) nucleic acids (NAs) are extracted from relatively large (~mL) volume sample lysates using an embedded porous silica glass fiber or cellulose binding phase (“membrane”) to capture sample NAs in a flow-through, filtration mode; (2) NAs captured on the membrane are isothermally (~65 °C) amplified; (3) amplicon production is monitored by real-time fluorescence detection, such as with a smartphone CCD camera serving as a low-cost detector; and (4) paraffin-encapsulated, lyophilized reagents for temperature-activated release are pre-stored in the chip. Limits of Detection (LOD) better than 10^3^ virons/sample can be achieved. A modified chip with conduits hosting a diffusion-mode amplification process provides a simple visual indicator to readily quantify sample NA template. In addition, a companion microfluidic device for extracting plasma from whole blood without a centrifuge, generating cell-free plasma for chip-based molecular diagnostics, is described. Extensions to a myriad of related applications including, for example, food testing, cancer screening, and insect genotyping are briefly surveyed.

## 1. Introduction and Overview

In the last decade there has been considerable interest and research in addressing the needs and opportunities for *on-site* Nucleic Acid-based Tests (NATs) [1,2,3,4,5,6,7,8,9,10,11,12,13,14,15,16,17,18]. These tests are also termed Nucleic Acid Amplification Tests (NAATs) when incorporating sequence-specific enzymatic amplification of nucleic acids such as PCR (polymerase chain reaction), or *molecular diagnostics* when used in a medical context. Such *in vitro* diagnostic tests implemented with low-cost, easy-to-operate, portable instruments and miniaturized sample processing/analysis devices can be used outside of medical laboratories for detecting pathogens in blood, urine, saliva and other sample types. NAATs provide more timely results compared to traditional culturing methods for diagnosis, and also work with the many pathogens that cannot be cultured. More generally, pervasive, cheap, and rapid NAATs will foster new paradigms for improved and sustainable medical care (e.g., *mobile healthcare* using smartphones) and new tools for research and discovery. For example, point-of-care (POC) diagnostics devices have been developed for quantifying HIV viral load with finger-prick blood samples. Closely related microfluidics technologies have been demonstrated for food and water safety testing [19,20], rapid genetic tests [21], environmental monitoring [22], surveillance for bioterrorism agents [23,24], cancer screening [25,26,27], assuring the health and hygiene of laboratory animals [28], analyzing biopsies [29,30], testing of livestock and pets for parasites [31], examining insects for genotype and infections [32,33], and first responder-administrated triage tests for stroke biomarkers [34].

To date, most rapid tests for biomarkers are immunoassays [35]. The lateral flow strip test, or immunochromatographic assay, such as found in home pregnancy tests, is an elegantly simple implementation of immunoassays employed to detect antigens or antibodies in urine, blood, food, and water. Molecular assays for DNA and RNA targets offer many important advantages over immunoassays, including greater sensitivity (typically by a factor of 1000 or more) and higher specificity. The high sensitivity is due to the ~million fold amplification of the target sequence. The amplicons can be conveniently detected with intercalating dyes or reporters that specifically label the amplicons, allowing measurements with relatively simple fluorescence detectors such as CCD cameras or photodiodes, or less commonly with electrochemical sensors. Further, unlike immunoassays which rely on monoclonal antibodies and may require weeks or months of development time, NAATs utilize nucleic acid oligos for hybridization or as primers for enzymatic amplification, which can be designed, synthesized, and validated in a short timeframe—in some cases as short as one week—once the pathogen has been at least partially sequenced [36].

While immunoassays can be performed on crude, unprocessed samples (e.g., whole blood or raw food) at room temperature, more elaborate sample processing and instrumentation is required for molecular diagnostics. In particular, (1) virons and cells must be lysed to release nucleic acid (unless cell-free NAs are the target); (2) substances, such as heme in blood specimens, that may inhibit downstream processes such as reverse transcription and amplification, must be removed; (3) amplification reaction temperatures must be closely regulated (typically within ±1 °C); (4) the target NA must be concentrated so to reduce amplification reaction volume to 10 to 50 µL in order to economize reagents and facilitate precise temperature control and uniformity; and (5) amplicon detection schemes should provide a statistically reliable means to discriminate between true and false positives, and true and false negatives. Thus, in general, although molecular diagnostics provide greater sensitivity and selectivity than immunoassays, they require more sample preparation, more instrumentation, more labor and expertise, and longer test times. Fortunately, NAATs can be implemented in a microfluidics system for manual, semi-automated, or fully-automated operation that can be readily adapted for point-of-care use. A broad aim of microfluidics POC efforts is to make molecular diagnostics nearly as easy to use as lateral flow strip immunoassays.

*Microfluidics* refers to various methods and technologies wherein small quantities (1 nL to 1 mL) of liquids are manipulated, processed, and analyzed using miniaturized, e.g., palm-sized, devices. In biotechnology, microfluidic components and devices have been demonstrated for, among other things, serial dilutions; cell fractionation, sorting, and enrichment; cell and virus lysis; isolation, concentration, and purification of nucleic acids; purification of proteins; immunoassays; reverse transcription; labeling of biomolecules with reporters; enzymatic amplification (e.g., PCR); electrophoresis; Micro total analytical systems (µTAS) combine these functions into an integrated sample-to-report device for applications in medical diagnostics; environmental, food, and water monitoring; detection of bioterrorism over conventional benchtop processing agents, and as tools for research, discovery, and quality assurance. The potential advantages of microfluidics include lower cost, faster results, effective containment of hazardous or infection material, reduced cross-contamination from other samples, better utilization of smaller samples sizes, reduced consumption of reagents and reduced generation of waste, portability, miniaturization, and ease of use for operation by minimally trained people.

Here, we review microfluidic “lab on a chip” (LOC) technology integrating sample preparation and analysis for point-of-care molecular diagnostics and other *on-site* NAATs. As such, microfluidic systems can support and complement microarrays. While microarrays offer a high degree of parallel processing for multiplex detection (hundreds or thousands of targets), microfluidics can streamline and automate the serial sample processing steps starting with crude, heterogeneous samples and yielding the requisite concentrated and purified nucleic acids for enzymatic amplification, microarray analysis, and sequencing. A 10- to 100-fold concentration of target for amplification is often needed. For example, the viral nucleic acid in 1 mL of blood plasma needs to be concentrated to a volume of about 10 µL for amplification. The “backend” functions of LOC molecular diagnostics systems which include amplification, labeling, and detection are well established. However, the “front end” functions of interfacing with the outside world through sample collection devices, as well as sample preparation and processing (lysis and NA isolation) of variegated heterogeneous samples are less developed. The “backend” functions are generic and largely independent of sample type and application, but the “front end” functions need to be tailored to specific applications (e.g., viral load measurements in blood *vs.* bacteria detection in food samples) according to the required sample size determined by the concentration of target and the sample and the desired Limits of Detection (LODs) [13]. Other factors related to sample processing include the need for and difficulty of lysis of tissue, cells and viruses containing the target NA, the presence of inhibitors intrinsic to the sample or added to the sample to effect lysis, and the stability of analytes, e.g., labile RNAs. Sample processing steps may also include deactivation of nucleases, proteases, infectious agents with reagents or heat.

Some degree of multiplexing is feasible in even simple microfluidic devices, and, as such, LOC devices may be suitable as low-cost, convenient alternatives to microarrays when multiplex detection of tens of targets suffices. This can be accomplished, for example, by incorporating multiple amplification reactors on a single chip, each containing primers for a specific target. The sample lysate is split and loaded on the several membrane/chamber channels, which can be imaged together using a CCD camera. Multiplexing in a single chamber would require probes specific to a target and with a unique fluorescence emission wavelength, combined with a color-sensitive imager. To support, supplement, or complement microarrays, standardized or customized microfluidic devices can be useful for rapid sample preparation and screening of samples where labile targets may suffer degradation (especially mRNAs) or low yield (e.g., microRNAs), quality control of reagents, detecting the presence of inhibitor substances and other interfering components, before the samples are subjected to more costly and time-consuming microarray analysis or sequencing. Further, knowledge of sequences gained with microarrays can be used to design amplification primers and probes for microfluidics-based NAATs.

## 2. Microfluidic Implementation of Nucleic Acid Isolation and Isothermal Amplification

Molecular diagnostics is comprised of the sequential steps of lysis (when the target nucleic acids are sequestered in cells or viruses); nucleic acid isolation (extraction, concentration, and purification of DNA and RNA from the lysate); reverse transcription for RNA targets, enzymatic amplification of target DNA or RNA with sequence-specific primers; and real-time or endpoint detection of amplification products. In the laboratory, these *unit operations* are typically carried out in benchtop instruments. First, the sample is incubated with lysing agents (salts, detergents, enzymes, chaotropic agents), sometimes supplemented with mechanical grinding, sonication, freeze-thaw, and heating, to release and solubilize nucleic acids. Next, nucleic acids are isolated from the lysate, commonly using a spin column kit for solid-phase extraction, where nucleic acids are captured on a silica membrane. Lysing agents such as chaotropic salts promote preferential binding of nucleic acids to silica glass or cellulose fibers of the porous membrane. The membrane is then washed with ethanol:water salt solutions to remove proteins and other debris. The captured NA is then desorbed and eluted from the membrane with water. At this stage, the captured NAs are concentrated and of sufficiently purity for enzymatic amplification, typically by PCR in a thermal cycler instrument. In the last five years, many new isothermal amplification methods, e.g., LAMP (Loop mediated AMPlification), helicase dependent amplification, NASBA (nucleic acid sequence-based amplification), and RPA (recombinase polymerase amplification) have gained prominence for POC diagnostics [17]. Traditionally, amplification products are detected and assessed by gel electrophoresis, but nowadays, real-time monitoring of the amplification process, using fluorescent intercalating dyes (SYBR^®^ green, SYTO^®^ green, Evagreen^®^) or molecular probes (e.g., Taqman^®^) is preferred due convenience and shorter protocols (obviating the need for post-amplification analysis), the feasibility of relative quantification, and lower risk of laboratory contamination by amplicons. Alternatives for detection of amplification products include lateral flow strips analysis of amplicons made with primers conjugated with antigens, or other real-time methods based on monitoring various products or by-products of amplification such as changes in pH, turbidity, electrical conductivity, phosphate or ATP concentration, some of which can be monitored by luminescence reactions using, e.g., luciferase/luciferin. Generally, this NAAT laboratory protocol requires six or more hours, and is carried out by skilled technicians in dedicated facilities equipped with hoods, centrifuges, temperature baths, and benchtop thermal cycler instruments. The labile reagents (polymerases, reverse transcriptases, proteases for lysis)—and often the sample too—need to be maintained in a cold chain, and the laboratory work area and procedures must minimize cross contamination of samples.

**Figure 1:**
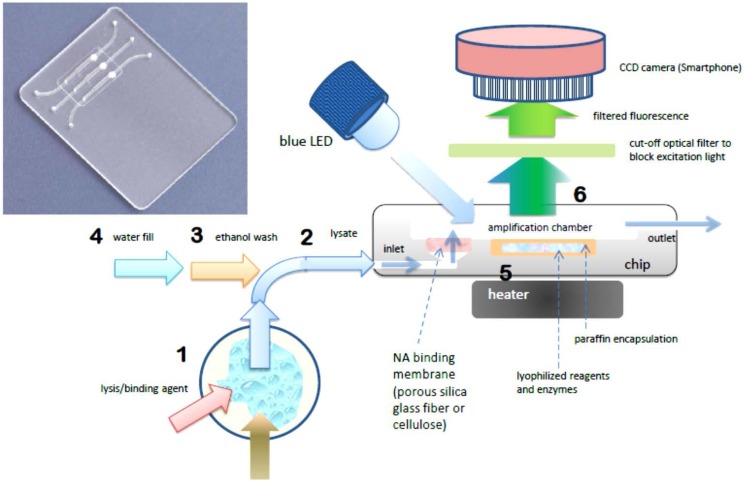
Chip using single-chamber for NA isolation, isothermal amplification, and real-time detection. Photo of chip with three parallel chambers for multiplex detection (inset). Molecular diagnostics process flow schematic (right): **1** Sample is mixed with lysis/binding reagent (guanidium HCl), **2** sample is injected into chip, **3** ethanol wash is injected into chip, washing NA (nucleic acid) capture membrane (silica glass fiber or cellulose disc embedded in chip), **4** chamber is filled with water and sealed. **5** chamber is heated to amplification temperature (65 °C), melting paraffin encapsulation layer and reconstituting lyophilized enzymes, reagents, and DNA intercalating fluorescent dye, **6** blue light excitation from LED generates fluorescent signal (proportional to amount of DNA amplicon) detected through filter (to block excitation light) by CCD camera (Smarthphone).

The foregoing benchtop procedure can be implemented with a credit-card sized microfluidic cassette “chip”, with total sample processing times of under one hour [18]. A photo of a representative chip is shown in Figure 1. The chip hosts three parallel reaction chambers for the isothermal amplification of nucleic acids. A porous cellulose (for DNA) or silica glass fiber (for RNA) “membrane” disc (~0.5-mm thick and 2 mm in diameter) is placed at the inlet of each chamber. During the sample loading stage, NAs are captured on the porous membrane solid phase, as sample lysate perfuses through the membrane. The NAs bound on the membrane are then washed with ethanol-based solutions to remove proteins and other contaminants. The chamber is then filled with water and heated to the amplification temperature, typically 60–70 °C, depending on the primer annealing temperatures and optimum temperature of the polymerase enzyme. During the heating to amplification temperature, the lyophilized reagents, enzymes, and dye that were pre-stored in the reaction chamber are released and hydrated. By encapsulating the reagents in paraffin that has a melting temperature below the amplification temperature, the stored reagents are shielded from the sample loading and wash solutions that flow through the chamber prior to amplification, and are reconstituted during the heated amplification step after the chamber is filled with water. The target NAs captured on the membrane are then amplified. RNA targets are simultaneously reverse transcribed into cDNA templates by reverse transcriptase, or an RNA/DNA dependent polymerase is used [37]. This approach avoids the need for a separate elution step as include in the benchtop spin column protocol. Specificity is dictated by appropriate design of the amplification primers. As with benchtop real-time PCR, the increasing amplicon concentration is monitored in real time during the amplification process. The fluorescence emission can be detected with an inexpensive USB microscope, photomultiplier, or smartphone (see Section 4). The amplification time required for the emitted fluorescence intensity to cross a specified threshold intensity is correlated with the concentration of target NA in the sample. The chip performs multi tasks to simplify its design, operation, and flow control and to reduce its cost. To summarize, the chip combines the following unit operations in a single reactor: (1) A porous silica or cellulose NA binding “membrane” is used to load sample lysate in a flow-through mode, allowing NA to be isolated from large sample volumes, such that the amplification volume is thus decoupled from the sample size, which is crucial for detecting low abundance targets. This feature is absent in many previously-reported POC NAAT devices; (2) *in situ* amplification of NA captured on the membrane, which eliminates the need for a separate elution step and simplifies flow control; (3) Isothermal amplification such as loop mediated isothermal amplification (LAMP), which simplifies the instrumentation compared to PCR and is less energy demanding than thermal cycling. LAMP appears more tolerant of inhibitors compared to PCR; (4) pre-storing in the amplification chamber paraffin-encapsulated, lyophilized amplification reagents (polymerases, reverse transcriptases, buffer salts, nucleotides, oligo primers, and DNA-binding dyes) further reduces the number of flow control operations, thereby simplifying the chip. Further, use of lyophilized reagents relieves both the need to add reagents at the time of use, and the need for any cold storage of reagents. As an added benefit, the method provides a “hot start” to amplification in that reagents are released only once the reactor has reached its operating temperature, reducing non-specific amplification. Moreover, it is feasible to store different reagents with different primer sets in separate parallel reactors, enabling the detection of multiple targets as well as providing control and calibration reactors; (5) Our chips enable real-time detection of the amplicon production, which provides more information than merely end-point detection, and allows one to terminate the test based on the target concentration, *i.e.*, when targets are available in high abundance, the test can be concluded in as little as 10–15 min; (6) The fluorescence emission can be detected with a CCD camera which readily enables concurrent monitoring of a large number of reactors and the utilization of smartphone cameras for cost reduction.

**Figure 2:**
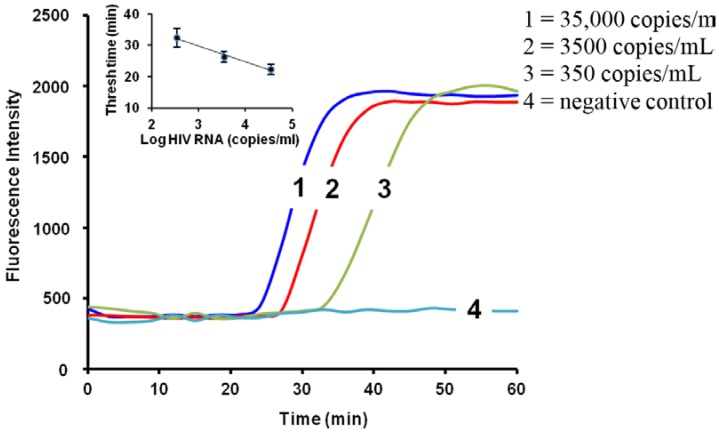
Real-time fluorescence amplification curves for HIV viral load measurements from plasma samples. The inset depicts the threshold time as a function of the log of target concentration [18].

As a specific application example, we used the chip described above to assess viral load in plasma samples (see below). Several hundred microliters of plasma were mixed with an equal part of lysing buffer (6 M guanidinium HCl), briefly incubated (~10 min), and pipetted into the chip, through the embedded nucleic-acid isolation membrane. Next, 100 µL of ethanol:water (50:50) was pipetted into the chip to wash the membrane. Finally, the chip amplification chamber was filled with water. The chip inlet and outlet ports were sealed with tape, and the chip was then inserted into a portable palm-sized instrument with a heated stage and mounting fixture for a cellphone. Figure 2 shows the results for plasma samples spiked with three different concentrations of HIV virons plus a negative control, indicating a LOD (limit of detection) of 350 copies per mL of plasma sample.

## 3. A New Method for Quantifying Nucleic Acids

**Figure 3:**
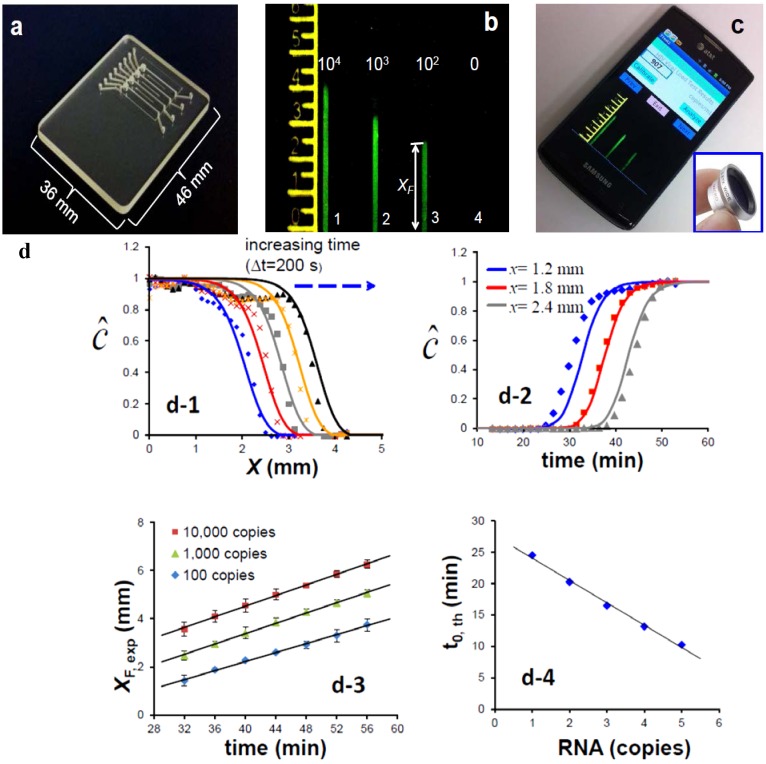
Nucleometer chip. (**a**) The chip having a similar structure to that described in Figure 1. The amplification-diffusion column is pre-filled with all the ingredients needed for the amplification process and a polymer to reduce diffusion rate; (**b**) As the target diffuses into the column it amplifies. After a short time that depends on initial target concentration, the column consists of two regions. A region in which the reaction has been completed (column of light) and a region in which the reaction has not yet started (dark region); (**c**) The test results can be monitored, documented, and transmitted with a smartphone. The front position of the fluorescence region is extended as a function of time and can be used to estimate target (NA template) concentration; (**d-1**) relative concentration of amplicon as function of distance along the conduit, as determined by fluorescence; (**d-2**) relative concentration of amplicon along the conduit, as determined by fluorescence, as a function of time; (**d-3**) distance along conduit (*X*_F_) as a function of time for different starting template concentrations (copies); (**d-4**) threshold time of fluorescence region extension *vs.* RNA template copy number [39].

**Figure 4:**
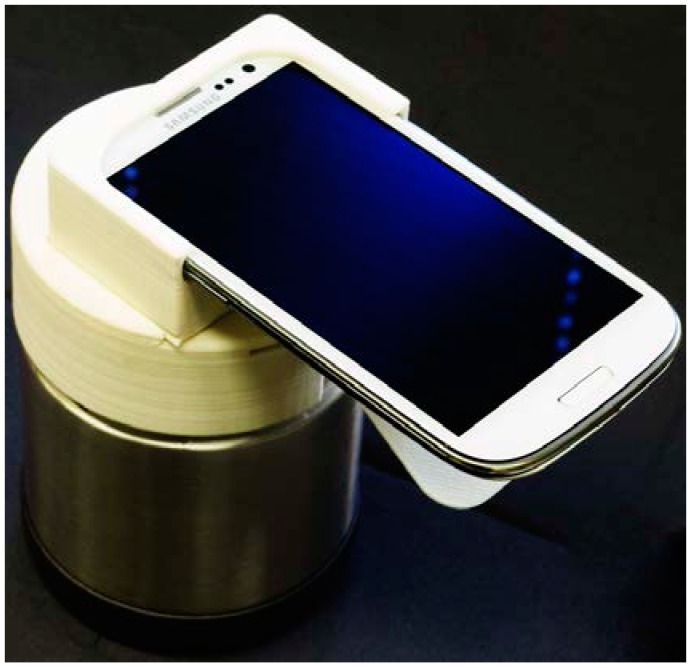
Smartphone mounted on portable instrument that provides the chip with temperature-regulated heating, optical excitation with a blue LED, a filter to block the excitation light from the smartphone camera and detection. The heating is provided with an exothermic reaction in which a magnesium alloy powder interacts with water. The temperature is regulated with a phase change material (PCM, e.g., paraffin) that acts as a ballast to maintain the reaction chambers at the PCM melting temperature, since excess heat production is absorbed as latent heat, maintaining the reaction chamber at the PCM melting temperature [42].

Alternatives to real-time monitoring of the amplification process for quantifying nucleic acids that are less demanding of imaging and computational capabilities could prove more amenable to low-cost POC diagnostics in resource-limited settings and may open new opportunities for NAATs for identification of bacterial flora and gene expression profiles. One such method that has been recently developed by our group consists of an amplification-diffusion conduit (ADC), which provides a spatial indication of the concentration of target nucleic acids in the sample (Figure 3) [38]. This “nuclemeter” chip, shown in Figure 3a, hosts four ADCs to illustrate a serial dilution effect of target concentration on device operation. Briefly, the ADC extends from the embedded silica membrane that captures NA, as described above, and which provides the source of template NA for amplification and diffusion. The ADCs are typically 330 µm wide × 330 µm deep × 12 mm long and are pre-filled with a stabilized gel (hydroxypropyl)methyl cellulose (HPMC) to retard diffusion and with all the ingredients needed for the amplification reaction, sans the target itself. The ADC is maintained at the amplification temperature (65 °C for the LAMP process). As the target diffuses into the conduit, it amplifies. Eventually, the amplification process runs its course (*i.e.*, saturates). After a short time from the beginning of the process, the reaction-diffusion conduit can be viewed as consisting of two regions: A region in which the reaction has been completed and a region into which the target nucleic acids have not yet diffused and the amplification reaction has not yet started. When an intercalating dye is used, the first region of the ADC, in which the reaction has been completed, emits fluorescence and appears as a column of light, while the second region, in which the amplification reaction has not yet occurred, appears dark (Figure 3b) which can be imaged with a Smartphone camera (Figure 3c). The two regions are separated by a relatively sharp reaction front. Once the initial reaction region has saturated, the reaction front moves with a constant velocity v (Figure 3d-1,d-2). That is, the position of the front is given by the equation *X*_F_ = *X*_0_ + *v·*(t − t_0_). *X*_0_ is the front location when the amplification process near the ADC’s entrance has saturated and *t* is time (Figure 3d-3. The magnitude of *X*_0_ depends on the concentration of the target nucleic acid in the sample. The greater the concentration of target molecules in the sample, the larger is *X*_F_. Figure 3d-4 depicts the position of the front as a function of target concentration in the sample at a given time. The reaction front velocity depends on the rate of the amplification reaction and the rate of diffusion and is typically ~0.1 mm/min. Since the position of the diffusion front at any given time can be correlated with the concentration of the target molecules in the sample (Figure 3d-4), it serves a similar role to that of the threshold time in real time machines. ADCs operating with known quantities of target molecules can serve to calibrate the length of the fluorescent column to enable quantification. The emission from multiple columns can be conveniently recorded with a smart phone camera.

## 4. Cellphone Detection of on-Chip Fluorescence

Microfluidic devices can leverage smartphone technology to image the chip and detect the increasing level of fluorescence due to the amplification process [39,40,41,42], as well as for computation and control functions, communication of test results (including GPS), data logging, and connect to sources of medical advisory information to the tested person. In one implementation [42], a blue LED (@ 20 mA current) excites the DNA-binding fluorophore, and the longer-wavelength fluorescence is detected by the CCD camera after optically filtering out the excitation light. The CCD camera of a smartphone proves to be an adequate, and widely-available, low-cost detector of green fluorescence (using SYTO green, EvaGreen™ dye, or similar DNA intercalating dyes) for real-time monitoring of amplification on the chip. The digitized image of the amplification chamber, generated by custom APP (mobile application) software, can quantitatively assess the increasing intensity of fluorescence during the course of amplification to determine a threshold time which correlates with the starting NA template concentration captured on the membrane. Further, many parallel chambers can be simultaneously imaged for multiplex detection, including several analytes, positive and negative controls, and calibration standards. Figure 4 shows a fixture for mounting the cellphone over the chip docked on a custom made, electricity-free heating stage.

## 5. Microfluidic Plasma Extraction from Whole Blood

**Figure 5:**
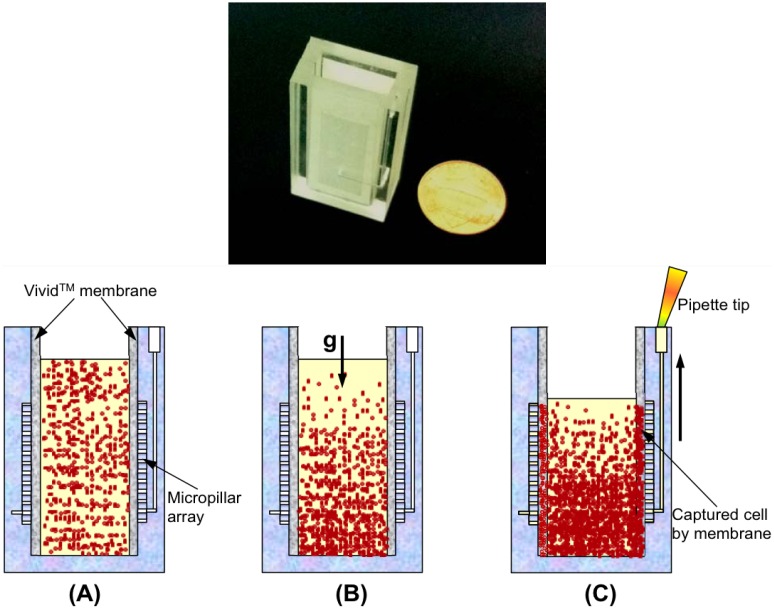
Miniature plasma extraction device that utilize sedimentation and filtration. Photo of the box model of the device (**top**). Processing stages (**bottom**): (**A**) loading of whole blood; (**B**) sedimentation of blood cells and filtration of plasma; (**C**) collection of plasma from side chambers [44].

Molecular tests for bloodborne pathogens are usually based on detection of NA biomarkers in cell-free plasma. Whole blood sample processing is problematic due to cells clogging the NA binding membrane, heme from lysis of red blood cells inhibiting enzyme action, and genomic DNA from white blood cells, for example, containing integrated retroviruses, confounding and complicating test results. Furthermore, prevailing standards for viral loads are based on virus counts in plasma rather than in whole blood. In a laboratory setting, the cell-free plasma component of blood is typically prepared as the supernatant of centrifuged (e.g., 10,000 rpms for 10 min) whole blood. Many POC venues do not have access to centrifuges, and therefore a simpler, instrument-free method for extracting plasma from whole blood samples is highly desired. Various microfluidic approaches for POC plasma extraction often involve supporting equipment such as electric motors and actuators, or vacuum pumps. Non-instrumented microfluidic devices rely on capillary flow or filtration, but these techniques either have low yield (volume of plasma extracted/volume of blood sample) or are limited to small blood sample sizes (~10 µL). In our work [43], we have developed a plasma extraction device based on combination of gravitational sedimentation and filtration through a porous membrane to achieve practical performance levels for field use (figure 5). Our device is a rectangular cartridge (2 cm × 1 cm × 4 cm) with a vertically oriented center chamber open at the top, and in which two opposing sidewalls featuring arrays of micropillars (300 µm × 300 µm in cross section, and 100 µm tall), machined or stamped, support polysulfone membranes. The membrane has asymmetric pores (larger diameter facing the center chamber) such that blood cells are gently trapped without lysis. The advantage of our approach is in allowing cells to sediment to the bottom of the chamber while plasma effuses through the vertical membranes, trickles trough the pillar arrays into trench channels, from where it can be collected by a pipette. The advantage of our approach is in reducing the amount of cells that come into contact with the separation membrane, thereby increasing separation membrane capacity. For example, 1.8 mL of whole blood loaded into this device yielded nearly 300 µL of plasma in 7 min. In a newer version of the device (not shown), we have nearly tripled the yield. An assay for heme in the extract indicated negligible lysis of red blood cells. Quantitative PCR of the plasma extracted from whole blood spiked with HIV indicated that the plasma is of sufficient quality for enzymatic amplification and that the virus recovery is over 80%. Figure 2 (shown earlier) provides amplification results of a serial dilution sensitivity data for HIV LAMP in the chip, using plasma samples extracted with our plasma separator as described above.

## 6. Chip Materials and Chip Prototyping

The microfluidic devices described above can be fabricated by several prototyping methods [44]. Indeed, this is one of the advantages of microfluidics: Custom design and rapid prototyping of components within a timeframe of one to two days is feasible. Chips are made as bonded laminate structures from plastic sheet materials, including PMMA (polymethylacrylate, “acrylic”), polycarbonate, polystryrene or cyclic olefin copolymer. The fluidic circuits are drawn using computer aided design (CAD) software (Solidworks™ or Autocad™). A middle layer is patterned with the fluidic circuit by a CNC milling machinine, a desktop engraver, or a CO_2_ laser cutter. Alternatively, the chip can be directly formed using a 3D printer with clear acrylic-like polymer alloys. The smallest feature size (*i.e.*, channel width) is about 0.3 mm. Smaller depths can be obtained with thin laminates. The component sheets are aligned and bonded using adhesives, solvent bonding, thermal pressure bonding, or ultrasonic welding. Both resistive heating or Peltier cooling modules can be used for temperature regulation, along with a thermocouple temperature sensors. The power consumption is on the order of 1 Watt. A commercial temperature controller or microntroller can be used to regulate the temperature, or even simpler analog electronics control circuits are sufficient. Reagents are from commercial LAMP kits (Eiken Chemical, Tokyo, Japan). Freeze-dried lamp reagents (including primers and fluorescent dyes) as lyophilized spheres can be preloaded into the chip during assembly to simplify operation.

## 7. Conclusions and Outlook for Near Term Applications

The feasibility and utility of microfluidic lab-on-a-chip implementations of component subprocesses for NAATs, including plasma extraction, lysis, NA isolation, amplification, and real-time detection and end-point quantification, have been well demonstrated for representative sample types and varied applications. POC devices and operation are significantly simplified by (1) a multifunctional reaction chamber that includes an embedded membrane for the capture of DNA and RNA, storage of reagents, and the *in situ* amplification of NA on the membrane which circumvents the need for a separate elution step and decouples sample size from amplification reaction volume; and (2) isothermal (constant temperature) amplification such as LAMP (Loop mediated amplification) with real-time detection, using for example, a smartphone CCD camera as detector. When blood samples are used, the hybrid sedimentation-filtration plasma extraction device can replace centrifugation enabling plasma separation from whole blood specimens at the point of care. The plasma separator can be integrated with the nucleic acid amplification chip for seamless operation. 

POC molecular diagnostics devices were first reported in the mid-1990s [45], single-chamber PCR chips made in glass or silicon. In the last twenty years, these chips have evolved to more viable polymer materials, have integrated sample prep functions, included on-chip storage of reagents and buffers, and accommodate low-cost detection schemes, such as smartphone cameras, and as such now offer convenient, low-cost diagnostics suitable for use outside of traditional clinical laboratories, including resource-limited settings throughout the world.

We also describe a new paradigm in target nucleic acid quantification through the use of a chip-based amplification-diffusion channel. This method facilitates end point detection and enables the estimation of target (NA template) concentration from the lengths of emitting columns, akin to reading temperature in a mercury thermometer from the length of the mercury column.

The various modules described herein can be combined into total microanalytical systems for sample-to-report POC diagnostics devices. In this review, we focused on NAAT of HIV in blood, suggesting the application of similar designs for other nucleic acid based tests where time, convenience, cost constraints would preclude the use of a microarrays, while still achieving multiplexing capability and quantification of both RNA and DNA targets with only modest or no instrumentation. We use a solid-phase extraction for NA isolation based on the common chaotrope-silica method used in commercial spin columns. Related NA isolation methods based on ChargeSwitch^®^ Technology (CST) and solid-phase reversible immobilization (SPRI) are also potential alternatives. These point-of care molecular diagnostics devices complement microarrays and provide an alternative to expensive laboratory-based instruments. As a comparison, commercial PCR-based and related systems, such as the COBAS AmpliPrep/Taqman HIV-1 (Roche), VERSANT HIV-1 RNA (Siemens), RNA QT (bioMerieux), and RealTime m2000 HIV (Abbott) can process close to 100 samples in a two-hour time span. Limits of detection typically range from 50 to 100 copies/mL plasma [46]. These instruments cost over $100,000 and their use is restricted to modern clinical laboratory facilities.
